# Contour Integration in Dynamic Scenes: Impaired Detection Performance in Extended Presentations

**DOI:** 10.3389/fpsyg.2017.01501

**Published:** 2017-09-05

**Authors:** Axel Grzymisch, Cathleen Grimsen, Udo A. Ernst

**Affiliations:** ^1^Department of Physics, Institute for Theoretical Physics, University of Bremen Bremen, Germany; ^2^Institute for Human Neurobiology, University of Bremen Bremen, Germany

**Keywords:** contour integration, feature integration, sustained attention, dynamic scenes, perceptual learning

## Abstract

Since scenes in nature are highly dynamic, perception requires an on-going and robust integration of local information into global representations. In vision, contour integration (CI) is one of these tasks, and it is performed by our brain in a seemingly effortless manner. Following the rule of good continuation, oriented line segments are linked into contour percepts, thus supporting important visual computations such as the detection of object boundaries. This process has been studied almost exclusively using static stimuli, raising the question of whether the observed robustness and “pop-out” quality of CI carries over to dynamic scenes. We investigate contour detection in dynamic stimuli where targets appear at random times by Gabor elements aligning themselves to form contours. In briefly presented displays (230 ms), a situation comparable to classical paradigms in CI, performance is about 87%. Surprisingly, we find that detection performance decreases to 67% in extended presentations (about 1.9–3.8 s) for the same target stimuli. In order to observe the same reduction with briefly presented stimuli, presentation time has to be drastically decreased to intervals as short as 50 ms. Cueing a specific contour position or shape helps in partially compensating this deterioration, and only in extended presentations combining a location and a shape cue was more efficient than providing a single cue. Our findings challenge the notion of CI as a mainly stimulus-driven process leading to pop-out percepts, indicating that top-down processes play a much larger role in supporting fundamental integration processes in dynamic scenes than previously thought.

## 1. Introduction

In a natural environment, the visual system receives a constant stream of dynamically changing and high-dimensional information which must be processed efficiently in order to create a coherent picture of our world. Throughout the past century several heuristics and mechanisms have been proposed to explain how the visual system undertakes such a complex computational task (see e.g., Heider, 1970; Gilbert and Li, 2013 for an overview). Gestalt psychologists were pioneers in describing the ways in which the visual system may group, distinguish and/or segregate different objects in a visual scene (Koffka, 1935). One of their proposed heuristics was the “Law of Good Continuation” (Wertheimer, 1923; Coren and Girgus, 1980), stating that visual elements following smooth global trajectories are perceptually grouped. Contour integration takes this notion and adds the requirement of local oriented elements being aligned in a collinear or cocircular fashion in order to be perceived as a global “contour.” Humans are remarkably efficient at performing the integration of local elements into global contours (Field et al., 1993; Kovacs, 1996), even when there is jitter in the alignment of the individual contour elements (Field et al., 1993), or if a section of a contour is occluded (Yin et al., 1997) as in the Kanizsa triangle (Wang et al., 2012). Functionally, contour integration allows for the identification of boundaries and paves the way for segmenting a visual scene into objects and background.

Several psychophysical (Kellman and Shipley, 1991; Mullen et al., 2000; Li and Gilbert, 2002), neurophysiological (Bosking et al., 1997; Gilbert et al., 2000) and computational studies (Li, 1998; Ernst et al., 2012; Singh et al., 2014; Sarti and Citti, 2015) have helped to create a comprehensive picture of contour integration encompassing different levels of understanding. From these studies we have learned much about which physical properties elements in a scene must have, and how they must be positioned in relation to each other, in order to induce the perception of a contour. For example, contour perception deteriorates with increasing degree of separation between elements (Mandon and Kreiter, 2005; Strother and Kubovy, 2006; May and Hess, 2008; Ernst et al., 2012) and with increasing deviation of element orientation from perfect alignment to the contour path (Bex et al., 2001). In addition, contours have been found to have a higher salience if element properties have a greater degree of similarity, e.g., if their spatial frequencies (Dakin and Hess, 1998; Persike and Meinhardt, 2015) or phases (Hansen and Hess, 2006) are identical. Taken together, these studies revealed a great robustness of contour integration against variations in a multitude of stimulus parameters. On a neural level, this finding is supported by strong correlates of contour integration in electrophysiological recordings, which emerge independently of the behavioral task (Bauer and Heinze, 2002).

Moreover, contour integration seems to be a fast process. Before being masked, stimuli need to be presented for only a few tenths milliseconds for a contour to be perceived. In particular, a stimulus presentation time of only 30 ms suffices for human observers (Ernst et al., 2012) to perceive contours with performances over 75% correct (2-AFC task), and for macaque monkeys (Mandon and Kreiter, 2005) to correctly discriminate contours with a performance of about 66% (in a task with a 25% chance level).

The picture emerging from these studies is that as soon as single contour elements match the criteria for good continuation and contour integration, contour perception appears to have a pop-out nature—suggesting neural mechanisms dominated by feedforward or recurrent integration of visual information which are barely influenced by cortical feedback or the actual cognitive state.

However, this picture changes when the psychophysics of contour perception is related to neural dynamics and anatomical structures in the brain. Both electrophysiological and imaging studies have successfully identified neural signatures of contour integration in human subjects (Altmann et al., 2003; Mathes et al., 2006; Mathes and Fahle, 2007; Mijovic et al., 2014; Volberg and Greenlee, 2014), in cats (Gilbert and Wiesel, 1990; Samonds et al., 2006) and in macaque monkeys (Li et al., 2006, 2008; Gilad et al., 2013). While association fields measured as firing rate facilitation and suppression in dependence on the spatial configuration of two line segments have essentially confirmed the law of good continuation (Kapadia et al., 2000), investigating the dynamics of contour integration has turned up some interesting findings. In early visual areas such as V1, responses to individual Gabor elements comprising a stimulus have been observed as early as 40–140 ms after stimulus onset (Gilad et al., 2013), whether these Gabor elements are part of a contour or not. However, firing rate increments in V1 induced by the alignment of individual Gabor elements comprising a contour have been observed almost exclusively at a later stage, approximately 150–250 ms after stimulus onset (Gilad et al., 2013). This delay suggests the involvement of higher visual areas in the process of CI since V1 circuits would respond much faster. Chen et al. (2014) showed that neural signatures of contour integration indeed appear earlier in area V4 than in V1, and were also accompanied by larger firing rate modulations in V4 than in V1. Similarly, Lee and Nguyen (2001) found earlier and stronger activity patterns in V2 than in V1 when rhesus monkeys were presented with Kanizsa figures arranged to form illusory contours. These observations of the temporal dynamics during CI present further evidence for the idea that higher visual areas are more strongly involved in CI than V1.

These observations have two important consequences: First, the notion of contour integration as a process not involving any feedback interactions has been challenged. Second, if integration is predominantly performed in areas such as V2 or V4, which are known to be strongly influenced by attention, then contour detection might more heavily depend on the current cognitive state than previously thought, especially in situations where contour integration is based on very weak or noisy sensory signals.

Taking together both physiological and psychophysical work, in terms of spatial aspects of various stimulus configurations we obtain a coherent account of contour integration. However, in the majority of these studies, stimuli containing contours were static, and often viewing time was limited by showing targets only briefly. Therefore, it is presently unknown how efficiently contour integration is performed in dynamic stimuli where contours can appear and disappear over an extended observation time. This question becomes even more relevant when considering that dynamic stimuli are a default situation, since in natural environments scenes are observed over an extended period of time, and image content often changes continuously.

In this study, we investigated a prototypical situation in which subjects have to observe a dynamic scene for an extended period of time. The task for our subjects was to detect the appearance of a contour which was embedded into an ensemble of oriented and slowly rotating Gabor patches. The contour could form at a random time in a random place in the stimulus display. Specifically, we contrasted situations in which the target was presented from a blank slate and the stimulus was presented for only 235 ms, with situations in which the stimulus had to be viewed for an extended period of time and the target formed after viewing the stimulus for a random period which could last up to 4 s.

First, we investigated how efficiently contour detection is performed under these two scenarios. Considering the reported “pop-out” nature of contour integration, one would expect very similar performance in brief and extended dynamic displays. However, on a perceptual level extended viewing is different from brief presentation, and hence extended viewing might be accompanied by changes in cognitive state with competing expectations forming and being evaluated against the dynamic visual evidence. Given that neurophysiological evidence locates contour integration in visual areas such as V2 and V4 (Anzai et al., 2007; Chen et al., 2014), one might expect that these changes in cognitive state interact with contour integration, thus potentially leading to very different performances in these two scenarios.

Indeed we found performance to be significantly different between a brief dynamic presentation condition and an extended dynamic presentation condition. In the extended condition contour detection performance was approximately 20% lower than it was in the brief presentation condition, and reaction times were approximately 270 ms longer. We addressed this observation in the second part of our experiment by cueing a contour position and/or a contour shape in order to investigate to what extent directing observer's focus to a position in space, and/or to a particular shape, can support contour integration in extended vs. brief presentation. In situations where detection performance is low, we quantified how much the different cues can improve contour detection, and thus, to what extent the different cues can restore contour detection performance. We expected to find a differential effect in brief and extended presentations, hypothesizing that the visual system may be able to better capitalize on the information provided by the cues in extended presentations as this case would allow for top-down effects to take place. Finally, our paradigm allowed us to combine spatial and shape cues in order to investigate whether cue combinations yield higher perceptual gains than single cues. Our results show that cues indeed increase perceptual performance, and they suggest differential effects of the single cues or cue combinations when comparing brief and extended presentations.

## 2. Methods

### 2.1. Apparatus

A CRT monitor (Chuntex Electronics, Ultra Screen VL950T) with a refresh rate of 85 Hz and pixel resolution of 1,152 × 864 (37.1 × 27.8 cm) was used to display visual stimuli. The stimulus ensembles were created in Matlab (Mathworks, Inc.) and rendered in real time with the Psychophysics Toolbox 3 (Brainard, 1997; Pelli, 1997; Kleiner et al., 2007) using a quadratic aperture of 19.7 × 19.7° of visual angle (864 × 864 pixels) of the whole screen. A gray scale correction was performed with a gamma-corrected linear staircase consisting of 255 steps ranging from 0.11 to 113.1 cd/m^2^. Observers viewed the screen binocularly at a distance of 80 cm in a room with attenuated light. Fixation control was performed by means of an eyetracker (SR Research Ltd., Eyelink II) at a sampling rate of 250 Hz and responses were provided by means of two custom-built response buttons with a temporal resolution of 0.1 ms. Head movements were restricted using a combination of a chin rest and a forehead rest.

### 2.2. Visual stimuli

**Figure 1:**
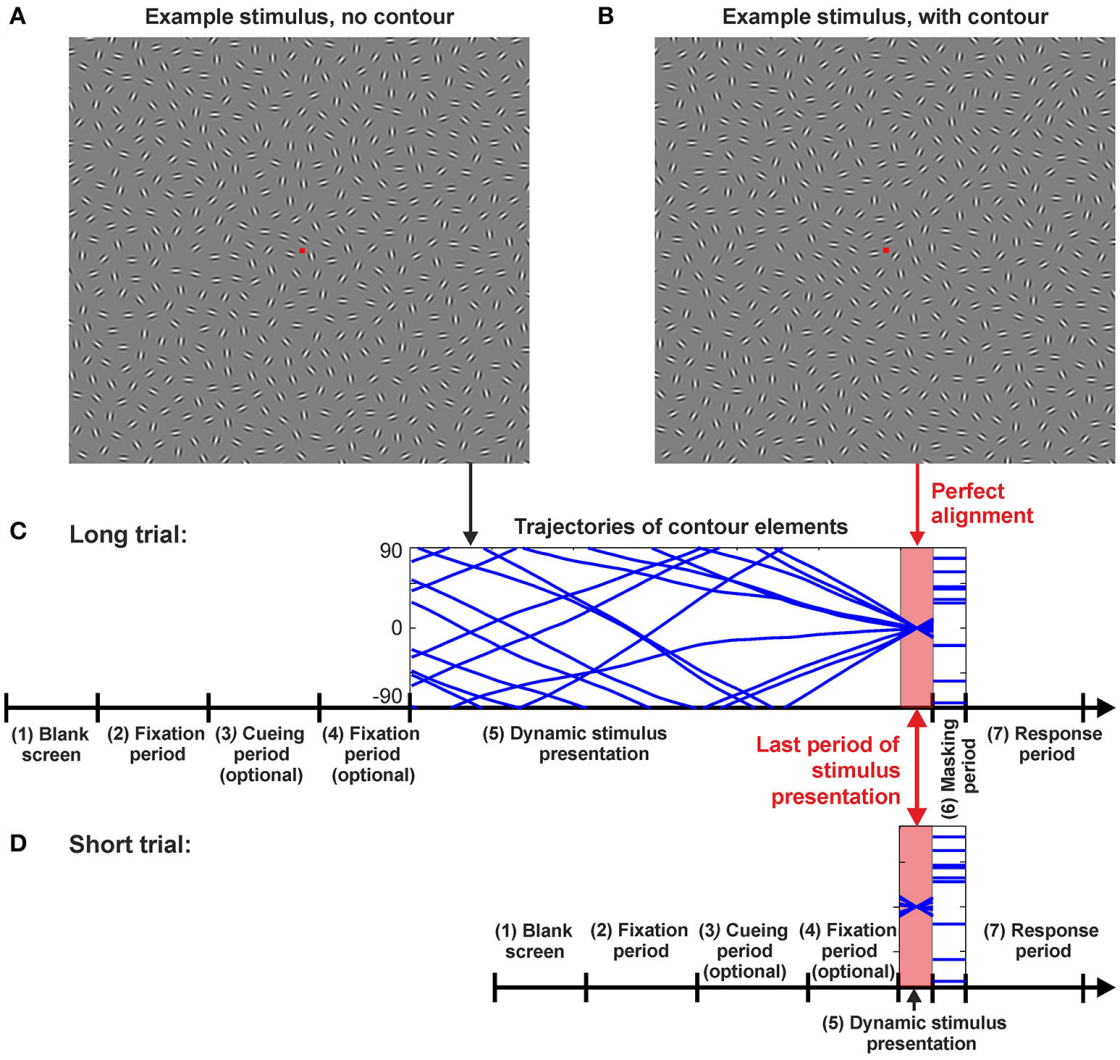
Example stimuli and trial sequences. **(A)** Example stimulus with a random arrangement of the Gabor patches. For most of the dynamic stimulus presentation time in the **Long** condition the rotating Gabors generated no meaningful figures, only toward the end of the stimulus presentation they aligned to form a contour as shown in example stimulus **(B)**. Here, the contour can be seen on the upper right quadrant, tilted at 45°. **(C) Long** and **(D) Short** trial sequences. The same sequence of events was followed for **Long** and **Short** trials, they only differed in the stimulus presentation times (SOAs) (period 5). The sequence of events for a trial was laid out as follows: (1) Blank screen for 1,176 ms; (2) Fixation spot for 2,353 ms; (3) Optional period—presentation of a cue in cued trials (see Section Methods); (4) Optional period—fixation spot for 588 ms in cued trials (see Section Methods); (5) Stimulus presentation. The time of stimulus presentation varied depending on the test conditions and it is refer to as *T* in the text; (6) Masking period for 588 ms; (7) Response period for 2,353 ms. For period 5 (stimulus presentation) either the **Peak** SOA (*T* = 235 ms), an adjusted SOA for the **Short** condition (*T*_*average*_ = 90 ms), or one of three different SOAs were used for the **Long** trials (*T* = 1,882 ms; *T* = 2,823 ms; or *T* = 3,764 ms). Presentation of these three distinct times in the **Long** condition were pseudorandomized. The perfect alignment period was identical for **Long** and **Short** trials as **Short** trials were realized by only presenting a section of a **Long** trial. Perfect alignment occurred shortly before the presentation of a mask (for **Long** trials *T*_*aligned*_ = *T*−117.6 ms and for **Short** trials *T*_*aligned*_ = *T*/2).

Visual stimuli comprised ensembles of oriented line elements (Gabor patches) which rotated in different speeds and in different directions during a trial. At a predefined time *t*_*aligned*_, a subset of these elements designated as the Gabours-in-contour (*G*_*c*_) aligned and formed a contour (the target—see Figure 1B). The task for the observers was to detect the contour and report whether it was placed in the left or right hemifield of the screen. Each stimulus contained exactly one contour. Contours were either right-oblique (45 ± 10°) or left-oblique (135 ± 10°), their global orientation was mainly straight, and they could appear in any of the four quadrants of the screen. Target contours in the stimulus ensembles were equally balanced amongst all of these defining qualities and pseudorandomized for presentation. For a sample of a typical trial in the **Long** timing condition (see **Baseline conditions**) please refer to the video provided in the Supplementary Materials.

#### 2.2.1. Single element properties and placement

Ensembles of Gabor patches were generated by placing elements on a hexagonal grid, and then subjecting them to a placement shifting process realized as a random walk with an additional constraint on minimal element distance of 0.21° of visual angle. Contours were embedded by selecting a set of 10 Gabor elements centered at a specific location near the center of one of the four quadrants and aligned with a specific orientation (left oblique or right oblique). Element orientations were set tangentially to a spline curve connecting the contour elements with minimal total curvature. Background elements were assigned random orientations. Each stimulus consisted of the ten Gabors designated as the Gabours-in-contour (*G*_*c*_) and an average of 550 Gabors designated as the background elements (*G*_*b*_), yielding a total of (on average) 560 Gabors. All Gabors were rendered with an even cosine phase and had a spatial modulation period λ of approximately 0.11° of visual angle. The standard deviation σ of the Gaussian envelope was set to 0.138° of visual angle, and the average separation between neighboring elements was 1.17°of visual angle.

#### 2.2.2. Stimulus dynamics

Gabor ensembles were presented for a time interval *T* during which elements rotated with different speeds and in different directions (clockwise or anti-clockwise). The rotation trajectory ϕ_*i*_(*t*) for each element *i* was generated from a random walk process on the angular velocities ω_*i*_(*t*) with a non-zero drift velocity ω_*r*_:

(1) $$\begin{array}{r} \tau \overset{\cdot }{\omega_{i}}\left(t\right)=-\omega_{i}\left(t\right)+\omega_{r}+\sigma_{r}N\left(0,1\right) \end{array}$$

(2) $$\begin{array}{r} \overset{\cdot }{\phi_{i}}\left(t\right)=\omega_{i}\left(t\right) \end{array}$$

For the parameters, we chose ω_*r*_ = 100°/s, σ_*r*_ = 120°/s, and τ = 3.3 s. To reduce higher salience of rotating elements near the fovea, rotation speeds were scaled with element eccentricity *e*_*i*_ according to the equation

(3) $$\begin{array}{r} \omega_{i}^{scaled}\left(t\right):=\omega_{i}\left(t\right)\left(1-\exp\left(-e_{i}/\lambda_{e}\right)\right)\; \end{array}$$

With λ_*e*_ = 4.2°, the rotation speed averaged approximately 20°/s near the fovea and 80°/s at more than 5° of visual angle eccentricity. After creating the trajectories, the initial phases ϕ_*i*_(0) for the *G*_*c*_ were shifted such that at a predefined time *t*_*aligned*_, the contour elements were perfectly aligned. This time was always close to the end of a trial. More specifically, for trials with *T* ≥ 235 ms, *t*_*aligned*_ was *T*−117.5 ms, and for *T* < 235 ms it was *T*/2. The maximal misalignment of the contour segments was approximately ±10° during a 235 ms period, thus, the perfect alignment of local elements in a contour to its global path was barely affected in the last 235 ms of the **Long** condition, or throughout the entire presentation of the **Peak** and **Short** conditions. Indeed, previous studies (Ernst et al., 2012) indicate that contour detection performance starts to drop only after about ±10° of jitter imposed on the alignment of individual contour elements. Hence, contours were maximally visible for the entirety of stimulus presentation in the **Short** and **Peak** conditions, and for at least 235 ms in the **Long** condition.

The parameter σ_*r*_ of the random walk was chosen such that after an average rotation of 180°, the spread of individual trajectories had a standard deviation of about 45° around this value, thus ensuring that perfect alignment of the 10 contour elements did not occur before the appearance of the target at *t*_*aligned*_. The chances of spurious contours of the same length as target contours appearing in the stimulus were negligible.

### 2.3. Procedure

The elapsed time of dynamic Gabor stimuli presentations containing the target contour had different durations *T* ranging from very short (a few 10 ms) to very long (more than 3 s) intervals (more information on the timing below). In a 2-AFC paradigm observers were instructed to indicate, as fast as possible, whether they saw a contour appear on the right or left hemi-field of the screen. Response buttons were held in each hand, and buttons were pressed with the corresponding thumb. Following a response observers were provided with auditory feedback indicating their performance in the trial. A tone with a high frequency was used for a correct response and one with a low frequency was used for an incorrect response. Observers were instructed to fixate on a central fixation square. If during periods 2, 3, 4 or 5 during a trial (see Figure 1) fixation was broken by means of an eye movement of amplitude larger than 3° of visual angle, or by an eye blink, a red screen was presented to the observer for 11.7 ms and the trial was aborted. Aborted trials were rescheduled at the end of a pre-set stimulus sequence. Since the contour always appeared shortly before the end of the presentation interval *T* (see Figure 1), dynamic Gabor stimuli were followed immediately by a mask consisting of static Gabor patches at the same positions but with random orientations. We employed this mask to prevent afterimages of the contour remaining on the retina (Bacon-Mace et al., 2005). In the following, we will refer to the time *T* when the dynamic stimulus was replaced by the static mask as the stimulus onset asynchrony (SOA).

#### 2.3.1. Training

All participants received a standardized four stage training (consisting of 24 trials at each stage), in ascending order of difficulty, prior to the start of the experiment. All four of these stages were done with a duration of the dynamic stimulus of *T* = 235 ms during which the alignment of contour elements could not deviate more than ±10° from the global path of a contour. In the first stage of training only contour elements were displayed, omitting the background elements and the mask. Observers were instructed to try to become familiar with the typical shape and location where a contour could form. Omitting the presentation of the mask and background elements helps to make the task easier to perform, increases performance, and thus aids in the training process. The second stage of training consisted of a typical trial with background elements, but again, without a mask. In the third stage of training full trial sequences with masks were presented without the added difficulty of having fixation control. In the last stage of training trials were presented in the same manner as it would be done in the experiment. After every stage of training participants were asked to report any difficulties they might have experienced, and were encouraged to ask any questions they may have developed; all doubts posed were clarified prior to the beginning of the experiment. Further training was provided at every stage of the experiment when the task was changed (i.e., when cues were introduced, see **Endogenousand ExogenousCues**). This training consisted of eight trials of the new condition presented prior to the first block of each condition. Prior to the start of training subjects were informed that target contours would only appear at the end of a trial. This was the case both for training trials and experimental trials.

#### 2.3.2. Comparing brief and extended presentations

In the first stage of our experiment, contour detection performance was tested under two different temporal conditions. All participants first performed the contour integration task at a relatively short stimulation time of *T* = 235 ms. Since previous studies (Braun, 1999) indicated that detection performance reaches a plateau from about *T* = 200 ms on, we used this **Peak** condition (total number of trials per observer *n* = 96, repeated on two consecutive days) to evaluate the ability of observers to perform the contour integration task. We did not explicitly test a static condition as our experience with static contour integration paradigms suggests that perceptual performance in our **Peak** condition is relatively similar to that of static contours presented with similar SOAs (Ernst et al., 2012). In the second stage of training observers performed contour integration in the **Long** condition (*n* = 96 repeated on two consecutive days), where three different stimulation periods were used (*T* = 1,882, 2,823, and 3,764 ms)^1^ in a random order. This procedure ensured that the observers could not predict the time when the contour would appear in the dynamic display, and thus had to sustain attention over an extended period of time. Figure 1 illustrates the stimulation sequence for all timing conditions. The stimuli in both conditions were derived from the same trajectories, however, in the **Peak** condition only the last 235 ms of a trajectory were shown.

#### 2.3.3. Establishing a baseline for cueing experiments

One goal of our study was to explore whether or not there are benefits for contour detection when cues about a target's position and/or shape are provided. In order to quantitatively compare improvements between brief and extended stimulus presentations observers had to start from the same baseline when no cues were given. As a baseline for each observer we chose their performance in the **Long** condition since it turned out to be much lower than in the **Peak** condition (see Results Section), thus offering more room for potential improvements.

In order to match an observer's performance in brief presentations to their performance in extended presentations we introduced the “**Short**” condition where *T* was adjusted for each observer individually so that contour detection performance was at a baseline level *p*_*Short*_ approximating their performance *p*_*Long*_ in the **Long** condition. This was done by an iterative Bayesian scheme: Since SOA is discretized in multiples of the frame rate *f* = 85 Hz, we initially selected a set of candidate frame numbers *k* spanning a range from *k*_*min*_ (minimal SOA *T* = *k*_*min*_/*f*) to *k*_*max*_ (maximum SOA *T* = *k*_*max*_/*f*). Starting from a uniform prior distribution, we iteratively computed the probability *P*(*k*) that a frame number *k* would give us the desired performance *p*_*Long*_. In each iteration, we sampled a test frame number *k*_*test*_ from *P*(*k*), presented a stimulus using the corresponding SOA, and recorded the observer's response. Taking into account the number of correct responses for each frame number, we then computed *P*(*k*) as the product of

1. The likelihood that the number of correct responses for *lower* frame numbers was obtained from performances *lower* than *p*_*Long*_
2. The likelihood that the number of correct responses for *higher* frame numbers was obtained from performances *higher* than *p*_*Long*_, and
3. The likelihood that the number of correct responses for *k* frames was obtained from a performance *p*_*Long*_

under the constraint of a 2-AFC paradigm (i.e., the underlying performances for all *k*'s between 0.5 and 1, and using binomial statistics). Finally, we normalized *P*(*k*) such that $\underset{k}{\sum }P\left(K\right)=1$. After a fixed number of trials, this procedure yields an estimate k^*^ = argmax_*k*_*P*(*k*) of the SOA $T_{Short}=k^{*}/f$ to be used to obtain a performance close to *p*_*Long*_. Observers with *p*_*Long*_ < 0.6 were excluded from this procedure and from any further experiments, since this value would indicate the observer did not perceive the contours in the **Long** condition. We always used a set of six candidate frame numbers *k*, starting from *k* = 5, *k* = 7, *k* = 8, or *k* = 9 for performance values of *p*_*Long*_ < 0.65, 0.65 ≤ *p*_*Long*_ < 0.7, 0.7 ≤ *p*_*Long*_ < 0.75, or 0.75 ≤ *p*_*Long*_, respectively, chosen from our previous experience on how performance depends on (short) SOAs (Ernst et al., 2012). After successful completion of this procedure, observers were tested in the **Short** condition with their individual recommended SOA *T*_*Short*_ < 235 ms with *n* = 96 on two consecutive days. Subsequently, *T*_*Short*_ was employed for all further stages of the experiment with brief presentation times.

#### 2.3.4. Endogenous and exogenous cues

In order to quantify the improvement in contour detection performance by directing an observer's focus to spatial and/or configurational features of the target, we employed cues that were presented prior to the dynamic Gabor ensembles. A **Shape** cue indicated whether a contour would be right-oblique or left-oblique, and a **Position** cue indicated whether a contour would appear in the upper or lower half of the screen. A combination (**Combination** cue) of these two cues was also used in order to evaluate whether the visual system is capable of combining cues in contour integration to further improve performance.

**Figure 2:**
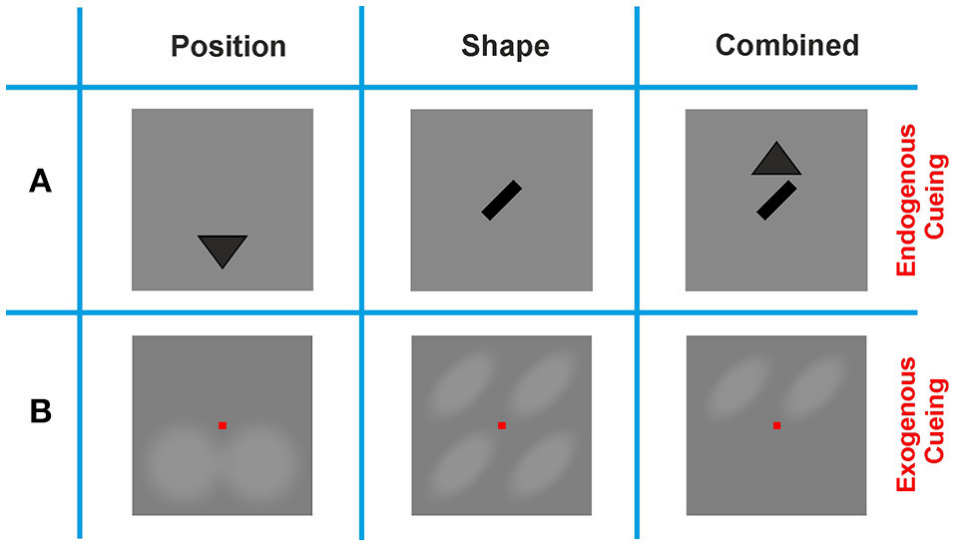
Cueing methods. **(A) Endogenous** cues provided the contour feature, or location information in a symbolic manner by presenting either a triangle indicating whether a contour would appear on the lower or upper half of the screen (**Position** cue), an oriented bar indicating the tilt of the contour (**Shape** cue), or a combination of these two cues (**Combination** cue). **Position** cues appeared either below or above the position of the fixation square, and **Shape** cues were centered in the middle of the screen. **(B) Exogenous** cues provided the same information in an explicit manner, by shading either the upper or lower section of the screen (**Position** cue), four oriented ovals at the possible locations where a contour could appear (**Shape** cue), or two oriented ovals at the possible locations where a contour could appear (**Combination** cue). Note that the scales of the **Endogenous** and **Exogenous** cues in this display are different: while **Exogenous** cues cover most of the stimulus display, **Endogenous** cues were much smaller and shown in the immediate vicinity of the fixation spot.

To evaluate the effects of different cueing methods we employed **Endogenous** and **Exogenous** cues. **Endogenous** cues gave semantic information only; by realizing the **Shape** cue as a left-oblique or right oblique bar of size 1.05° of visual angle at the center of the screen, and by realizing the **Position** cue by filled triangles of the same size, pointing upwards or downwards above or below the center of the screen, respectively (Figure 2). **Exogenous** cues were realized by increasing the brightness of specific regions on the screen, thus directly indicating potential contour positions and/or contour shapes. The regions where brightness was manipulated formed a translucent outline and were blended in and out of the uniform gray background in a smooth manner. This presentation was chosen in order to avoid sharp on- or offsets which might have disturbed observers' focus, or might have driven them to generate a saccade and break fixation. For each of the two cueing methods, and for each of the cueing conditions (**Position**, **Shape**, and **Combination**), each observer performed *n* = 96 trials. For the baseline condition (**No-Cue**) *n* = 192 trials per observer were performed. If the performance obtained in the **No-Cue** condition turned out to be larger than the required baseline performance (e.g., through on-going perceptual learning during the progress of the experiment), the session was repeated with a shorter SOA on a subsequent day.

#### 2.3.5. Block design

To avoid potential memory/adaptation effects from a fixed presentation order of the different temporal and cueing conditions we employed block designs for the experimental sessions investigating **Exogenous** and **Endogenous** cueing. For **Endogenous** cues we pseudorandomized the presentation of **Position**, **Shape**, and **Combination** cues and **Long** and **Short** SOAs. Prior to each experimental session a schedule was generated for each observer detailing the order of presentation of the three cues, and the order of **Long** and **Short** SOAs. **Long** and **Short** blocks were always alternated for each of the three cues, and the order of presentation for the cues and the SOAs were pseudorandomized to ensure that approximately an equal number of participants would be presented with the **Long** or the **Short** timing condition first, and that the order of cue presentation was not the same for all participants (i.e., the order of cue presentation was circularly rotated by one position for each subsequent participant in the order **Combination**, **Position**, **Shape**). For **Exogenous** cues we employed a fixed block design for the order of cue presentation. This was done with the aim of easing a participant into the experiment by presenting the cues in a descending order of difficulty. The order of cue presentation was fixed to the following sequence: **Combination**, **Position**, **Shape**, and **No-Cue**. The order for the different SOAs in the **Short** and **Long** timing conditions was pseudorandomized in the same manner as it was done for **Endogenous** cues.

### 2.4. Participants

Using **Endogenous** and **Exogenous** cues, the full experiment stretched over four sessions: **Endogenous** cues, first day (session #1), **Endogenous** cues, second day (session #2), **Exogenous** cues, first day (session #3), and **Exogenous** cues, second day (session #4). 10 participants (7 females) served as observers for sessions #1 and #2, and 9 (6 female) of the initial 10 participants served as observers for sessions #3 and #4. After initial analysis of the behavioral data, part of sessions #3 and #4 had to be repeated with a different SOA *t*_*Short*_ in the **Short** condition (see “Perceptual Learning” in Results Section). In this second round of testing, only 7 of the original 10 observers could participate, hence two extra observers were recruited for this section of the experiment to have a complete data set of 9 observers (4 females). The age range of observers was 24–41 years. Observers were paid 10 Euros per hour for their participation and were informed about the purpose of the experiment prior to the experiment. All observers reported normal or corrected to normal vision. All participants provided written consent according to the regulations of the local ethics committee and according to the World Medical Association Helsinki Declaration. The study was approved by the local ethics committee on the 6th of May 2014.

## 3. Results

Paired sample tests were used at all stages of the analysis with a significance level of α = 0.05 unless otherwise stated.

### 3.1. Contour detection is much harder in prolonged presentations

**Figure 3:**
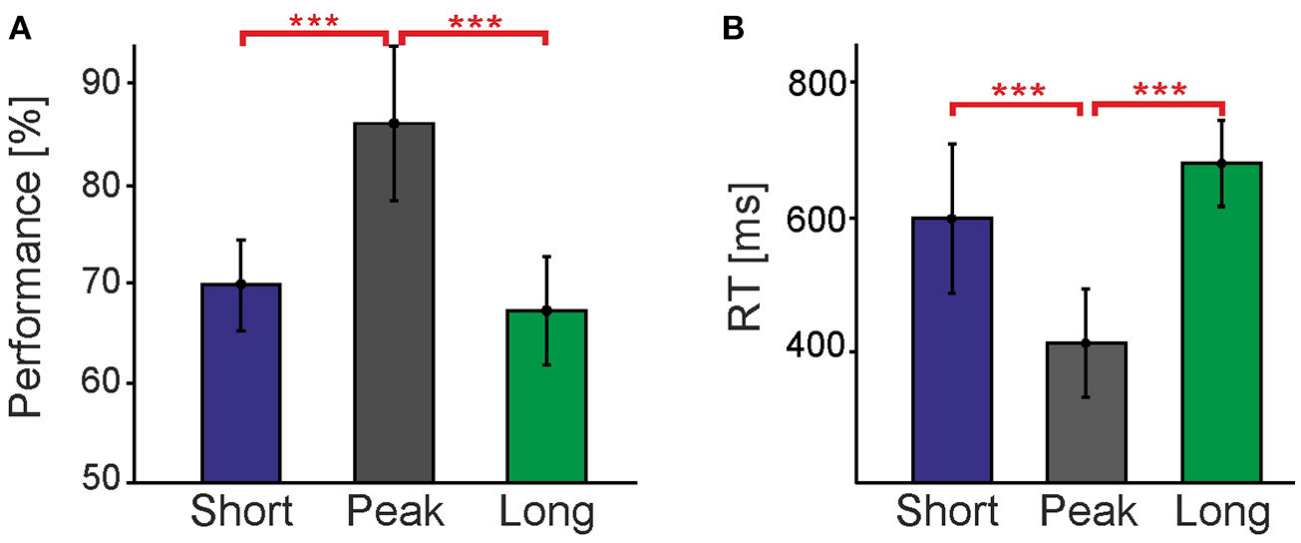
Comparison of different timing conditions without cues. **(A)** shows performance and **(B)** reaction times for the **Short**, **Peak**, and **Long** timing conditions without cues. Vertical bars indicate standard errors. The **Short** data consists of trials performed with distinct *T*s for each individual observer obtained with the staircase procedure in order to match their performance in the **Long** condition. Asterisks represent the level at which these conditions were found to be significantly different (^***^p < 0.001). Performances and RTs in the **Short** and **Long** conditions establish the baseline for the cueing experiments shown in the subsequent figures.

Figure 3 compares contour detection performances and reaction times (RTs) in the **Peak** and **Long** conditions. All performances were significantly higher than chance level (50%), showing that observers could successfully perceive contours in all conditions. However, it turned out that contour detection is much harder in the **Long** condition, with performance being 18.8% lower [*t*_(8)_ = 10.3, *p* < 0.001] and RT being 268.8 ms longer [*t*_(8)_ = −9.6, *p* < 0.001] than in the **Peak** condition. This is a surprising result considering that: (a) the last 235 ms-long period of the stimulus containing the target contour was exactly identical in both the **Peak** and **Long** conditions; and (b) that typically approximately 100 ms exposure to a (masked) stimulus is already sufficient to obtain 75% correct contour detection performance, and that performance plateaus at about 200 ms of exposure to said stimulus (Braun, 1999). Apparently, the dynamic history of the stimulus in the **Long** condition preceding the formation of the target contour induces a strong suppression of our ability to detect contours.

By separating detection performances for the three different presentation intervals (1,882, 2,823, and 3,764 ms) contained in the **Long** condition we investigated whether the observed suppression develops slowly over time. The performances for the three intervals were almost identical at 67.1, 67.5, and 67.5%, respectively, and not significantly different from each other [*F*_(2)_ = 0.01, *p* > 0.05]. The same result was obtained for RTs [808.0, 774.9, and 745.9 ms, respectively, with *F*_(2)_ = 2.99, *p* > 0.05]. Consequently, we can conclude that suppression of contour detection emerges on a fast time scale and is already fully developed at about 1.9 s after stimulus onset.

This observation of a much lower contour detection performance in extended presentations of dynamic stimuli stands in stark contrast to the apparent ease with which our visual system performs in dynamic visual environments, relatively independent of the length of exposure. One putative explanation is that in extended presentations, top-down processes become more important and help to enhance perception and compensate for this pronounced suppression—possibly at the expense of not being able to focus on a scene as a whole, but only to a small part of it (Simons and Rensink, 2005).

Following this hypothesis, we next quantified to which extent cues of particular contour shapes and/or a particular contour positions can improve contour detection and hence reduce the suppressive effect. In particular, we suspected that top down processes might act *differently* in brief and extended stimulation contexts, being potentially *more effective* in the **Long** condition given that the timing of this condition may allow for top-down mechanisms to come into play. To investigate these questions, we first had to find a method to reduce performance in short stimulus presentations to the same level as in the **Long** condition, without changing the actual target stimulus, in order to establish an identical baseline allowing comparison of potential improvements. Standard methods to reduce performance such as decreasing stimulus contrast (Kingdom et al., 1992; McIlhagga and Mullen, 1996; Hall et al., 2014) or imposing orientation alignment jitter (Ernst et al., 2012) are problematic since they would physically change the target stimulus and potentially involve different neural mechanisms in contour integration. Instead we chose a reduction of stimulus presentation time before appearance of the mask (the SOA/Period 5 in Figure 1) as our control parameter. Previous work has demonstrated that reducing the SOA to values below 100ms leads to a monotonically decreasing contour detection performance (Li, 1998; Ernst et al., 2012).

Using a staircase procedure (see Section Methods) presentation times were reduced to, on average, *T* = 90 ms (*S*.*D*.: 21.38 ms) around the point of perfect alignment in the new **Short** condition. The **Long** and **Short** conditions were not found to differ from each other, neither in terms of performance [*t*_(8)_ = 1.2, *p* > 0.05] nor RT [*t*_(8)_ = 0.60, *p* > 0.05], thus establishing the desired baseline to quantify performance gains (Figure 3).

#### 3.1.1. Cueing improves detection performance in all conditions

**Figure 4:**
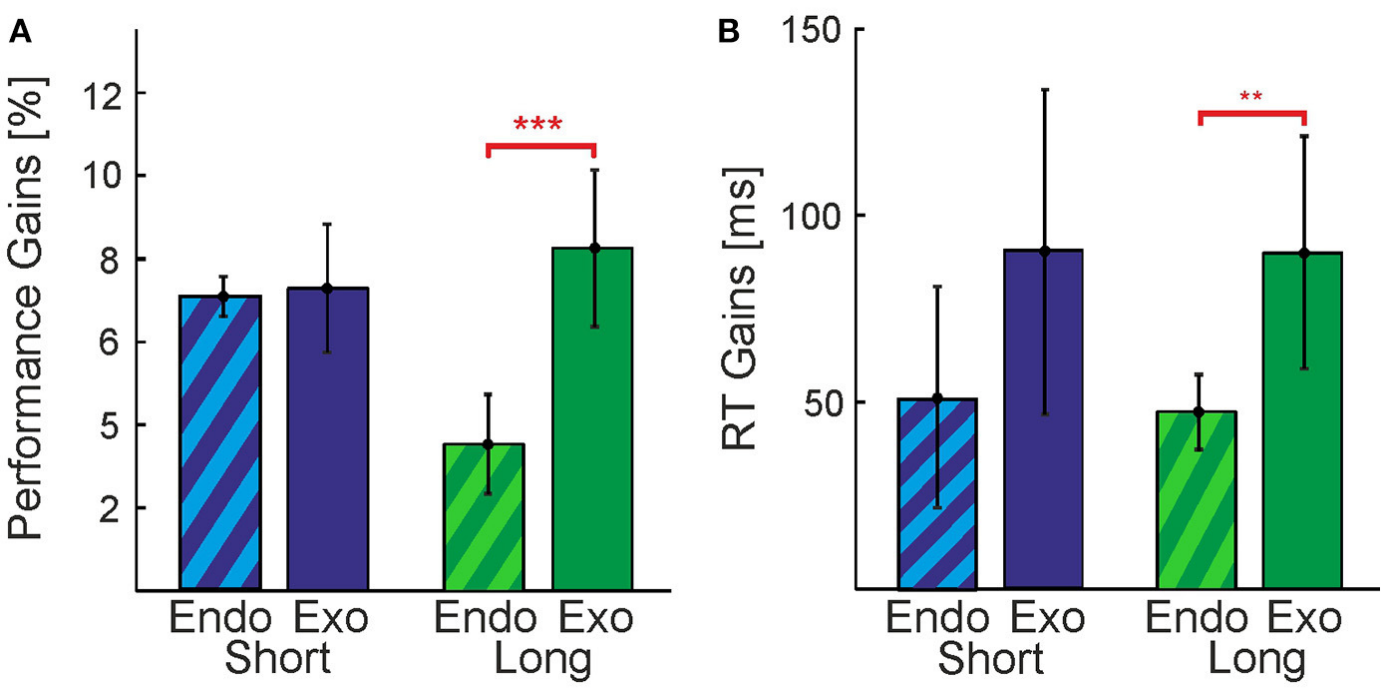
Comparison between **Endogenous** and **Exogenous** cues. Perceptual gains expressed as **(A)** increases in performance and **(B)** decreases in reaction times. Vertical bars indicate standard errors. Data was collapsed over all cueing conditions (**Position**, **Shape**, **Combination**) for **Endogenous**(candy stripes bars) and **Exogenous** cues (solid bars). Gains were calculated by subtracting the baseline condition (**No-Cue**) from the average over all cueing conditions. Differences between cue types in the **Short** timing condition were not significant. Significant differences at levels ^***^p < 0.001 and ^**^p < 0.01 were found for performance and reaction time gains, respectively, in the **Long** condition.

In our experiments we used **Exogenous** and **Endogenous** cueing methods, providing direct visual or indirect symbolic information about the nature of the contour that is hidden in the stimulus, respectively (see Section Methods and following paragraph). Although both **Exogenous** and **Endogenous** cueing formally reduce the search space by the same amount, the quality of the provided information is different and might be easier or harder to use. We first analyzed the overall effectiveness of the cueing methods (**Exogenous** vs. **Endogenous**) with non-paired *t*-tests. In order to perform this analysis we collapsed the data of the three cueing conditions (**Position**, **Shape**, and **Combination**). In general, we observe a pattern (Figure 4) of **Exogenous** cues providing greater gains in performance and larger reductions of RTs for both, the **Long** and the **Short** conditions. This indicates that **Exogenous** cues were more effective for our purposes, possibly because they were more explicit in indicating a contour's shape and/or position than the abstract symbols used as **Endogenous** cues. A clear exception is seen in Figure 4A for the case of performance in the **Short** condition [*t*_(52)_ = 0.88, *p* > 0.05]. In the **Long** condition the gain in performance and reduction in RTs for **Exogenous** cues was found to be significantly higher than that observed for **Endogenous** cues, *t*_(52)_ = 6.32, *p* < 0.001 and *t*_(52)_ = 3.41, *p* < 0.01, respectively. From Figure 4B we would expect to also find a significant effect for the difference in gains of RTs for the **Short** condition, however, this was not the case [*t*_(52)_ = 0.56, *p* > 0.05]. A clear outlier is seen in the data for this condition, if removed the difference in gains between the direct and indirect cues for this condition almost doubles from 39 to 71 ms.

In summary, we found that contour detection in our task is improved by both **Exogenous** cues and **Endogenous** cues, in terms of performance and RTs. However, we see smaller effects using **Endogenous** cues in most cases, and there is much more variability (noise) between individual observers. It seems that the visual system can make better use of **Exogenous** cues, possibly engaging top down processes in a more efficient manner to improve perception. Consequently, for the remainder of this section we choose to focus on **Exogenous** cues only, since the statistical power provided by the **Endogenous** cueing method for assessing the *differences* between particular cueing types (**Position** and **Shape**) and their combination is very low.

#### 3.1.2. In extended presentations, cue combinations are more efficient than single cues

In order to cue contours, we introduced a cue at the position of the contour (upper or lower hemifield, **Position** cue) and a cue on the shape of the contour (oblique left or oblique right, **Shape** cue), see Methods Section Figure 2B. There are 8 possible combinations of shape and position for a contour (4 positions × 2 shapes, disregarding the additional jitter/uncertainty on the exact position and shape), thus each cue reduces this number by a factor of two. Since the cues provide independent information, they could also be combined (**Combination** cue), leaving only two possible combinations of shape and position for a contour appearing in a stimulus.

**Figure 5:**
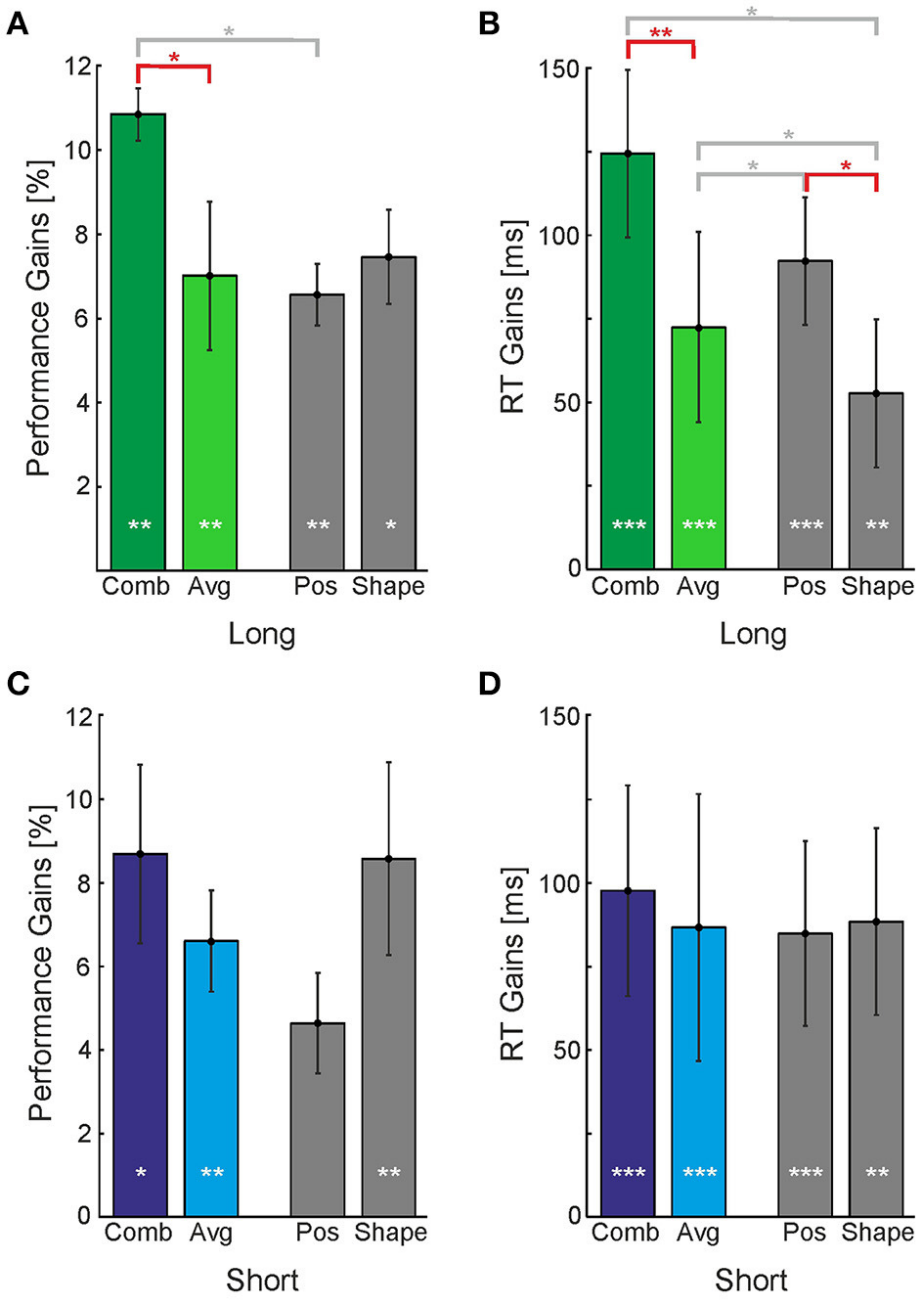
Perceptual gains provided by individual and combined cues. Perceptual gains expressed as **(A,C)** increases in performance and **(B,D)** decreases in reaction times, for the **Long (A,B)** and **Short** timing conditions **(C,D)**. Vertical bars indicate standard errors. Gains were calculated by subtracting the **No-Cue** baseline from the corresponding performances or RTs. Gray bars represent single cues (**Position** and **Shape**) whose average is displayed in the colored bar labeled *Avg*. White asterisk inside the bars represent the level at which the perceptual gain was significantly different from baseline. Asterisks above the horizontal bars represent the level at which two conditions were found to be significantly different (^*^p < 0.05; ^**^p < 0.01; ^***^p < 0.001). Horizontal bars indicating comparisons discussed in the text are displayed in red color.

We first collapsed the data of the **Position** cue and **Shape** cue in order to evaluate whether the **Combination** cue has a special advantage when compared to the average gains provided by single cues (colored bars in Figure 5). We found that in the **Long** timing condition performance and RT gains were greater for the **Combination** cue than for the average of the single cues [Figures 5A,B, *t*_(8)_ = 2.95, *p* < 0.05 and *t*_(8)_ = 3.46, *p* < 0.01 for performance and RT, respectively]. This was not the case for the **Short** timing condition [Figures 5C,D, *t*_(8)_ = 1.15, *p* > 0.05 and *t*_(8)_ = 1.15, *p* > 0.05 for performance and RT, respectively].

In the **Long** condition, the **Position** cue yielded a greater reduction in reaction times than the **Shape** cue [*t*_(8)_ = 2.52, *p* < 0.05], but it yielded no difference for performance. In the **Short** condition, Figure 5C indicates a reversal of this pattern: While there is no difference in the RTs, the **Shape** cue seems to provide a greater increase in performance than the **Position** cue. However, this difference of about 4% is not statistically significant. When compared to the **No-Cue** condition, however, both the **Position** and **Shape** cues yielded improvements in performance and reductions to reaction time for **Long** and **Short** presentation times. These results indicate that different cues might act in a different manner in short and extended presentations, however, there is too much variability among individual observers and sessions to make a strong point out of this observation.

Note that with the fixed order of cue presentation employed for **Exogenous** cues we sought to obtain a lower-bound estimate for the real performance gain without perceptual learning effects. We thus scheduled the cueing conditions in descending order with respect to expected performance gain (the order was **Combination**, **Position**, **Shape**, and **No-Cue**). As a consequence, the reported differences between the **Combination** and single cue conditions are a conservative estimate of the real differences; these differences would be larger if one could subtract the perceptual learning effect.

#### 3.1.3. Perceptual learning is faster for short presentations

**Figure 6:**
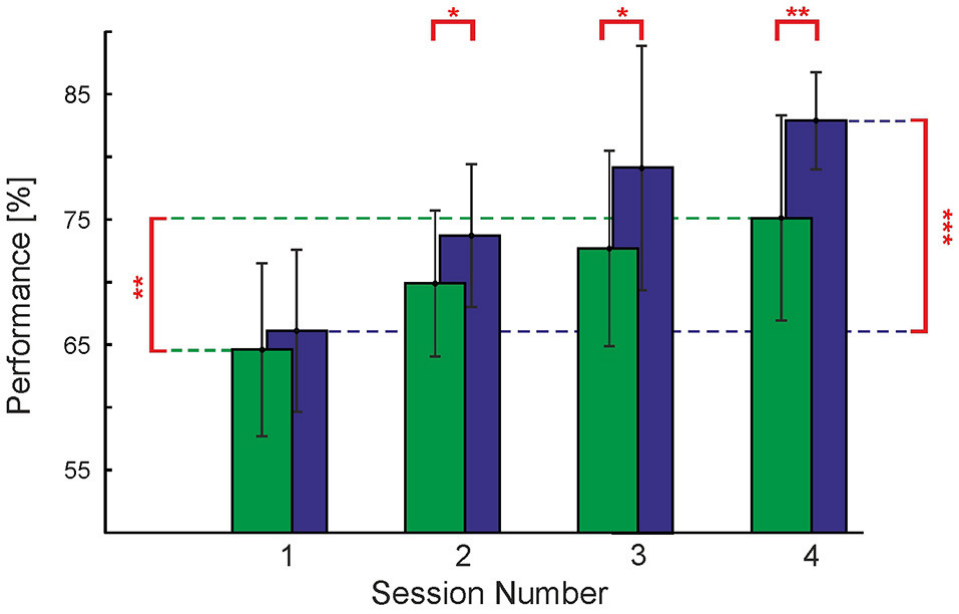
Perceptual learning. Contour detection performance and standard deviation (vertical bars) for the **Short** and **Long** timing conditions without cues, plotted over the subsequent four experimental sessions. Asterisks represent the level at which experimental sessions were found to differ from each other (^*^p < 0.05; ^**^p < 0.01; ^***^p < 0.001). Note that the actual time between experimental sessions varied among observers.

We also checked if contour detection performance increases over time, as it has been observed in previous studies (Schoups et al., 2001; Li et al., 2008). We assessed average behavioral performance over sessions taking place on subsequent days of experimentation in the order: (a) **Endogenous** cues, first day, (b) **Endogenous** cues, second day, (c) **Exogenous** cues, first day, (d) **Exogenous** cues, second day; see Methods Section. We found that throughout the testing sessions of both, **Endogenous** and **Exogenous** cues, observers' performance increased to a large extent. Focusing on trials using no cues, for the **Short** timing condition we can see an increase in performance of 16.8% from the first day of testing in the **Endogenous** cues experiment (i.e., the first session for each observer) to the second day of testing in the **Exogenous** cues experiment (i.e., the fourth session for each observer)—see Figure 6. This increase in performance was found to be statistically significant [*t*_(8)_ = 6.74, *p* < 0.001] with paired *t*-test. An increase in performance over the same time period was also seen in the **Long** timing condition. Here the increase was smaller, 10.5%, however, it was also found to be statistically significant [*t*_(8)_ = 2.41, *p* < 0.05] with a paired *t*-test.

This difference in learning rate between the **Short** and **Long** timing conditions has one important consequence: at the beginning of the first session, which used the **Endogenous** cues, we carried out a staircase procedure to find the observers' SOA which matches the performance of the **Short** timing condition to that of the **Long** timing condition (see Section Methods). At the end of this procedure we checked that performances in this condition, without cues, were approximately equal (64.6 and 66.1% for **Long** and **Short** timing conditions, respectively); a paired samples *t*-test with α = 0.05 did not reveal these two conditions to be statistically distinct. However, already on the second session (second day of testing **Endogenous** cues) there was an increase in performance of 5.3% for the **Long** condition and an increase of 7.6% for the **Short** condition. Although the corresponding difference of 2.3% is small, it is statistically significant [*t*_(8)_ = 2.85, *p* < 0.005]. As can be appreciated from Figure 6, the rate of improvement in the **Long** and **Short** timing conditions differed sufficiently throughout the four sessions to yield a difference of 7.7% between the two timing conditions on the second day of testing **Exogenous** cues, even though we started with a small, non-significant difference of 1.5%.

## 4. Discussion

In summary, we have found that extended presentations of dynamic stimuli affect the process of contour integration in an unexpected manner. Prolonged presentations lasting from 1.9 to 3.8 s (**Long** timing condition) lead to large reductions in detection performance when compared to presentations lasting about 235 ms (**Peak** timing condition). In both conditions the final 235 ms period in stimulus presentation containing the target contour was identical, hence the drop in performance was clearly a consequence of the dynamic stimulus shown prior to the moment of perfect alignment, and not a consequence of the rotation *per se*. Furthermore, we could show that cueing/attending a particular contour shape or position in the visual field can partially compensate for this effect and enhance contour detection in dynamic stimuli. In order to also quantify the effects of cueing in short stimulus presentations we first decreased SOA until contour detection performance matched baseline performance in extended presentations, thus creating the **Short** timing condition. Subsequently, we found similar improvements in performance when providing cues in the **Short** condition, indicating that independently of stimulus presentation time, attention can support contour integration to a similar extent. In addition, in the **Long** timing condition it appears that the visual system could make better use of cue combinations. Finally, we observed perceptual learning effects from testing block to testing block, which lasted weeks^2^, suggesting that functional changes must have taken place in the visual system leading to an improved contour detection performance. This improvement was clearly stronger for brief stimulus presentations. In the following, we will discuss our findings in more detail.

The stark reduction in performance for extended stimulus presentations was unexpected since contour integration has been shown to increase monotonically with increasing SOAs and to require only 60 ms to be reliably performed in macaque monkeys (Mandon and Kreiter, 2005) and only about 100 ms to achieve the same performance in human observers (Ernst et al., 2012). In both, **Peak** and **Long** timing conditions, there was a relatively ample window of time when the contour was almost perfectly aligned. In our stimulus the maximal rotation speed of elements at more than 5° of visual angle eccentricity was 80°/s, thus the maximal misalignment of the contour segments was ±10° during the final 235 ms period in both conditions. A jitter of ±10° is known to barely affect performance (Field et al., 1993), and indeed performance in the **Peak** condition was relatively high at about 85% correct responses. In the **Long** condition, shortly before the appearance of the target contour, the visual system must therefore have been in a state which severely impairs our ability to detect contours. In dynamic scenes this finding implies that contour integration might have a much smaller importance for visual perception and might require a much stronger support by other feature integration processes than previously thought. Furthermore, performance in the **Long** condition was initially low (about 65%), thus some observers did not have a conscious percept of the contours—this effect might have implications for other cognitive processes which we will discuss later (e.g., perceptual learning).

There might be several possible causes for the stark drop in performance, mainly: (a) neural adaptation processes, (b) perceptual hysteresis, (c) an increased attentional load or attention fatigue in the **Long** timing condition, (d) the effects of transiently enhanced neural activity immediately after stimulus onset, and/or (e) temporal and recurrent processes in visual perception. To elaborate:

- Adaptation is a phenomenon which is ubiquitous in neural systems: adaptation currents in neurons lead to a decreasing firing rate in response to a constant input current (Kohn, 2007), recurrent inhibition provides normalization of neural responses (Carandini et al., 1997), and synaptic resources deplete over time (Tsodyks et al., 1998). Stimulating the same spatial receptive fields may have led to adaptation in the retina or in the LGN, which may have been inherited by areas in visual cortex performing contour integration. On the cortical level, Carandini (2000) has shown that there are cellular mechanisms acting in individual cortical neurons which are responsible for adaptation processes after exposures to visual stimuli lasting several seconds, and that these can lead to impairment of our perception of subsequent stimuli. Motion adaptation effects which impair our perception have also been well documented and are attributed to a reduction of responsiveness in cells tuned to a certain aspect of a stimulus (Anstis et al., 1998). Hence, the extended presentation of rotating Gabor patches in the **Long** condition may have lead to decreasing firing rates and thus—assuming a constant background noise level—to a decreased ability to detect contours after a few seconds of presentation. However, one also has to take into account that Gabor rotation speeds were very slow compared to the time required for adaptation. Assuming adaptation takes place on a timescale of 50–100 ms, and a full rotation of a Gabor element takes at least 500 ms, orientation-selective neural populations in V1 would have almost fully recovered when a Gabor rotates through their preferred orientation a second time.
  Our observation that top-down processes improve performance can be explained by electrophysiological findings (Galashan et al., 2013) showing strong firing rate increases caused by attention even if the corresponding neurons are in a sustained state of low activation after prolonged stimulus exposure. Such increases in activity are also compatible with psychophysical studies (Yeshurun and Levy, 2003; Yeshurun and Marom, 2008) observing that attention increases the perceived duration of a stimulus while decreasing temporal resolution.
- Perceptual hysteresis, which has been defined as the tendency to stabilize a percept (Schwiedrzik et al., 2011), may be another explanation for the low performance in the **Long** condition. Perceptual hysteresis is different from adaptation: fMRI studies with human observers found that when a subject experiences hysteresis, ventral visual areas, superior parietal areas, and the frontal cortices tend to have a high degree of activation (Kleinschmidt et al., 2002; Schwiedrzik et al., 2012). In contrast, when observers experience adaptation in a similar task, areas V2 and V3 in the visual cortex tend to increase their activity (Schwiedrzik et al., 2012). In our case, in the **Long** condition percepts not related to the target contour might have emerged during extended stimulus presentation. Putative percepts could include proto-contours (i.e., contours of two or three accidentally aligned Gabors), or Gabor configurations with other regularities having a certain salience (i.e., star-like pattern). In the **Peak** condition, percepts other than the target contour itself would not have emerged because the contour was present from the beginning of the dynamic stimulation period and by construction it was the most salient feature. Furthermore, the short SOA of only 235 ms barely provides the necessary time for stable percepts to emerge.
- The required attentional resources to successfully perform the contour integration task in the **Long** timing condition may have been higher than in the **Short** timing condition, since the **Long** condition demands observers to engage in the task for several seconds. Fisher (1984) and Joseph et al. (1997) have shown that performance in visual search tasks is modulated by the number of features to which an observer must attend to in order to perform the task correctly, and by the rate of stimulus presentation. In our experiments the number of features which required attention did not change between the timing conditions. However, it is possible that the stimulus presentation time acted in an analogous manner to the stimulus presentation rate in Joseph et al. (1997). If this is the case, this would explain the low performance in the **Long** timing condition. Furthermore, the **Long** timing condition is particularly demanding in terms of attentional load. In this condition observers have to closely monitor the whole stimulus and are not allowed to move their eyes over an extended period of time. This is a situation in which attentional fatigue might have occurred, which would have led to a narrowing of the spatial focus of attention. Subsequently, and throughout the remaining trial, an observer's attention might have shifted to different areas of the stimulus—either to systematically scan the visual field with the now smaller attentional “spotlight,” or automatically drawn to salient features such as the chance alignment of a few Gabor patches emerging in different positions in the visual field (“protocontours”). As a consequence, the focus of attention of our observers could not have been at the location where the target appeared and hence they might have failed to detect it correctly. As the **Peak** condition consisted of a brief presentation of the stimulus (235 ms), shifting of attention to a different location in the stimulus is unlikely to have occurred.
- Performance could have been higher in the **Peak** timing condition due to the enhanced transient neural activity caused by the abrupt change from a blank screen to a screen full of Gabor patches (Figure 1, periods 4 and 5). It is well known that a transient in neural activity occurs at stimulus onset (Jonides and Yantis, 1988; Visscher et al., 2003). Most importantly, the transient goes along with a decrease in the Fano factor (Galashan et al., 2013), which implies an increase in the signal-to-noise ratio and hence enhanced information processing capabilities. In contour integration, one electrophysiological study even demonstrated that the initial transient of a V1 neuron's response is higher if the stimulus element inside its receptive field is part of a contour (Bauer and Heinze, 2002). In our case, contour integration in the **Peak** timing condition might have well profited from such an enhanced transient. The transient would of course also be present at the beginning of the stimulus presentation period in the **Long** timing condition, however, it typically lasts only about 50–100 ms (Galashan et al., 2013), and contours appeared in the **Long** timing condition no earlier than about 1.7 s after stimulus onset.
- In the **Long** condition one can think of the irrelevant dynamic stimulus prior to *T*_*aligned*_ as a forward mask. Forward masks have been shown to affect many perceptual tasks such as change detection (Wutz and Melcher, 2013) and feature integration (Herzog et al., 2001, 2003), thus the appearance of the target contour could have been missed by the observers due to this effect.
  One could also think of the dynamic stimulus prior to the appearance of the contour in the **Long** condition as a continuously evolving noise signal. This signal is potentially integrated over time, as shown in Burr and Santoro (2001), Melcher et al. (2004), and Drewes et al. (2015). Most interestingly, Burr and Santoro (2001) demonstrated that this time scale is limited to about 2–3 s, implying that detection performance of a target presented with an increasing SOA decreases until it reaches a plateau at an SOA of about 2–3 s. The situation is akin to the **Long** condition in our experiment, in which observers were subjected to a “noise” signal for at least 1.9 s before they were presented with the target (in the **Long** condition three different presentation intervals were used, 1,882, 2,823, and 3,764 ms, with the contour reaching the point of perfect alignment 235 ms before the end of these intervals). Given that a plateau is already reached at about this time, this would explain why we observed no further decrease in performance between 1.9 and 3.8 s.

EEG data from a recent study (Castellano et al., 2014) suggests that in extended viewing conditions, contour-related neural activity emerges very slowly. The authors considered a similar paradigm to ours (Grzymisch et al., 2013), in which contours formed dynamically by Gabor elements rotating into alignment after two seconds into a trial, and stayed in alignment for about one second before the contour dissolved again. Neural signatures of contour integration became significantly different from activity related to stimuli not containing a contour only after 150–250 ms, and needed as much as 400–600 ms to fully develop. In our case, the contour was present for maximally 230 ms until masked, thus terminating these slow processes far before they could reach maximum salience. This is further indication for contour integration in extended presentations being dominated, or even suppressed, by other on-going processes in the visual system.

Contour integration is subject to perceptual learning (Li et al., 2008; Gilbert et al., 2009). We observed perceptual learning in both **Long** and **Short** conditions. When participants are first presented with the stimuli typically used in psychophysical contour integration studies they often find it hard to detect contours, and their performance is significantly lower than after several hours of training. This might be due to the artificial nature of the stimuli used in experimental settings; typically stimuli are created in a manner which allows researchers to precisely control the parameters of interest and exclude all other cues. In our experiment we ensured that the only feature which could indicate the presence of a contour was alignment between the individual Gabor elements. However, in natural images there is usually a combination of features (e.g., color, texture, etc.) which indicates whether an element belongs to a given figure or object. Thus, it seems that the visual system of an average person might not be accustomed to detecting contours in the same manner, and especially with so few cues, as it is forced to do so in psychophysical experiments.

We also observed that it is not only harder for the participants to detect contours in extended dynamic presentations, but that the perceptual learning which occurs under this condition is not as pronounced as that seen for brief presentation times (see Figure 6). There are two possible scenarios explaining this finding, namely that asymptotic performance in **Long** and **Short** conditions are identical, but learning rates are different, or that learning rates are identical, but asymptotic performance is different. Since we only have four data points describing the temporal evolution of the performance over experimentation time (Figure 6), we can not decide between these possibilities. One reason supporting different learning rates might be our observation that in extended presentations, contours are not always consciously perceived. If perceptual learning takes place through reinforcement, then in such a situation there is no error signal that the visual system could use to improve stimulus processing, thus slowing learning progress. An alternative scenario supporting identical learning rates with different asymptotic performances would emerge if in extended presentations, neural activity is lower (adaptation) or the “noise” level is higher (as it would be if there is interference from other on-going computational processes).

Our results suggest that to achieve a high contour integration performance in dynamic scenes, support from top-down processes is needed. This finding is in agreement with physiological studies showing that top-down influences from higher visual, or other cortical areas strongly support contour integration (Li et al., 2008; Mijovic et al., 2014). Initially, we did not expect to find such a large difference in the **Long** and **Peak** conditions as contour integration is typically described as a robust process occurring mainly in early visual areas (Li, 1998; VanRullen et al., 2001; Hess et al., 2003), and as such it should be predominantly driven by the current stimulus and less so by the current behavioral state. From our study we can not conclude whether attention or other top down processes are independent of contour integration, simply acting as an amplifier on top of a contour integration process performed in an early visual cortical area, or if these processes interact with contour integration, actively boosting the linking process of visual features. The perceptual learning we observed throughout our experiments support the suggestion that contour integration involves networks in the extrastriate cortex and not just early visual areas. We make this claim since the perceptual learning we observed is distinct from that shown to take place in V1. In V1 perceptual learning is location-specific (Shiu et al., 1992; Schoups et al., 2001) and requires extensive training over about 2,000–5,000 trials (Schoups et al., 2001). In our experiment perceptual learning took place although the location where contours could appear varied significantly (four quadrants and two orientations, with some freedom in the exact position where a contour could appear in the quadrant), and we had a much lower number of trials (in total, each subject was exposed to about 1,500 trials, however, since the location and orientation of the contours the subject saw was evenly distributed amongst the four quadrants and two orientations the relevant number of trials for perceptual learning would approximately be 200). In summary, we speculate that top down processes invoked by external cues always play a role in contour integration when the task is difficult—only when a contour is in a pop-out, or static configuration, it appears that bottom-up processes suffice to perform efficient contour integration.

In conclusion, to study contour integration in dynamic scenes we employed a novel experimental design (Grzymisch et al., 2013; Castellano et al., 2014) which disentangles contour integration from neural processes linked to stimulus onset transients (such as enhanced activity). This approach paves the way for future studies to answer the question of why contour integration becomes so difficult under extended viewing conditions. In particular, we suggest to record from neurons in areas V1/V2 and V4 in order to distinguishing between feedforward/recurrent and top-down processes. This would allow us to explore the questions of how Gabor rotation activates orientation-selective neurons in orientation hypercolumns, whether the rotation of Gabors causes strong adaptations of neural responses, and how response variability scales with adaptation state and presentation time. Furthermore, by observing neural signatures of attentional states in V4 it will be possible to quantify how an attentional state changes over time, and to relate fluctuations in changing cognitive states to behavior (i.e., successfully/unsuccessfully detected contours).

## Author contributions

AG and UE contributed to the design, implementation and execution of the experiment. CG contributed during the design and execution phase. The data analysis, visualization of data, preparation and writing of this manuscript was done by AG and UE.

### Conflict of interest statement

The authors declare that the research was conducted in the absence of any commercial or financial relationships that could be construed as a potential conflict of interest.

## Footnotes

^1^We originally designed stimuli to be shown at a refresh rate of 100 Hz, but decided to use 85 Hz instead. For this reason, presentation intervals often have “peculiar” values such as 1,882 ms (this value would have been 1,600 ms at 100 Hz).

^2^The testing periods between the **Endogenous**(first two testing blocks) and **Exogenous** cues experiments (last two testing blocks) varied from a few days to several weeks between observers. After realizing that observers had difficulties in using the **Endogenous** cues we decided to improve our paradigm, thus, the first observers who were tested in the **Endogenous** cues experiment had to be called back to be re-tested in the **Exogenous** cues experiment, and for some observers this took several weeks.

## Supplementary material

Supplemental data comprises four video files showing the following experimental conditions:

Video 1 Complete trial from Peak condition, with added markers indicating contour location.

Video 2 Complete trial from Peak condition.

Video 3 Complete trial from Long condition, with added markers indicating contour location.

Video 4 Complete trial from Long condition.
